# Many More Dentists Needed

**Published:** 1913-07-15

**Authors:** Alfred P. Lee


### MANY MORE DENTISTS NEEDED.

BY ALFRED P. LEE, D.D.S.

Once a year at about this time several thousand young men anticipating graduation from dental colleges are brought face to face with the serious question of determining upon locations for practice. Many fortunate ones are not called upon to make this decision, circumstances such as the sharing of practices of fathers, entering offices as assistants, or succeeding elderly practitioners, relieving them of any anxiety in this direction. But by far the majority must look to the establishing of new practices, and it is but natural that the law of supply and demand should enter largely into the making of such decisions.

But so many elements render this law of supply and demand an uncertain one that statistics in reference to population and number of practitioners can only be considered in a general way. For example, some States show a large population as compared to the number of practicing dentists, but it should be remembered that many of these localities contain a relatively large alien or negro population, of which only a small proportion seek dental aid. References to other States may show that exactly reverse conditions obtain, and while these sections may appear to be overcrowded from a dental standpoint, they may, in reality, offer lucrative fields for young practitioners.

As a matter of fact, the whole situation, viewed from the standpoint of dentistry as a money-making vocation, may be considered as exceedingly hopeful for many years to come. Statisticians assure us that not enough dentists are being produced annually to make up for the increasing population. When, taken with this, it is considered that a larger proportion of the population is each year becoming educated to the needs and refinements of correct dental attention, it is readily seen that the shortage of dental practitioners may become a serious matter.

When the question of raising the dental course of study from three to four years was under discussion at the recent meetings of dental teachers at Pittsburgh, the supply and demand phase of the situation was presented by several of the delegates. Dr. C. R. E. Koch, of Chicago, who, as a historian, has given this subject no little attention, said: "If they (the dental colleges) do not produce a greater number of dentists with average capacity and average ability to serve the average people, they mistake their calling. * * We have this condition now: That we have not, with all the efforts of all these schools, since State boards have eliminated from admission to practice men who have not entered the profession through dental school doors, produced dentists enough to take the place of those who annually retire, and that in spite of the fact that our population is increasing (I refer only to the United States but I presume the same thing applies to Canada)—in spite of the fact that our country is growing at the rate of one million a year, who need, on an average of twenty-five hundred of population for one dentist, an *increase* of four hundred dentists per year. In addition to that fact we have the magazines, the public press, the public schools, all teaching the importance of oral hygiene—the fact the the mouth is the port of entry of the health of the body. * * *"

The writer is informed that several men of our profession have been making investigations as to the number of dentists in active practice in the United States, and that these investigations have led to the startling discovery that fewer men are practicing dentistry to-day than there were two years ago.

All this may present itself as an economic problem rather than the offering of a suggestion as to locality for practice, but it certainly shows that the dental ranks are far from being overcrowded, and that there will be lucrative practices in abundance for many years to come.

We publish herewith a table showing the population, number of dentists and number of people to each dentist in the United States. The population figures are from the census of 1910, while Polk's Register for 1912 is authority for the dental figures.

From other sources we find that each physician has in the United States 640 people, while each dentist has 2,366, or nearly four times the number as has his medical brother. It may be argued, and with some truth, that of the 640 all will at some time require medical attention, while a fair proportion of the dentist's 2,366 will never consult him,or at least only for such operations as extractions. But granting the strength of this argument, it will still appear that the opportunities for the dental surgeon are decidedly more hopeful than for the practitioner of medicine.

| State | Population | Dentists | People to each Dentist |
|---|---|---|---|
| Arizona | 204,354 | 64 | 3,193 |
| Arkansas | 1,574,449 | 359 | 4,386 |
| California | 2,377,549 | 1,903 | 1,249 |
| Colorado | 799,024 | 492 | 1,624 |
| Connecticut | 1,114,756 | 611 | 1,824 |
| Delaware | 202,322 | 80 | 2,529 |
| District of Columbia | 331,069 | 342 | 939 |
| Florida | 752,619 | 273 | 2,757 |
| Georgia | 2,609,121 | 630 | 4,141 |
| Idaho | 325,594 | 176 | 1,850 |
| Illinois | 5,638,591 | 3,203 | 1,760 |
| Indiana | 2,700,876 | 1,245 | 2,178 |
| Iowa | 2,224,771 | 1,198 | 1,858 |
| Kansas | 1,690,949 | 834 | 2,028 |
| Kentucky | 2,289,905 | 786 | 2,914 |
| Louisiana | 1,656,388 | 454 | 3,648 |
| Maine | 742,371 | 403 | 1,842 |
| Maryland | 1,295,346 | 578 | 2,241 |
| Massachusetts | 3,366,416 | 2,060 | 1,634 |
| Michigan | 2,810,173 | 1,421 | 1,972 |
| Minnesota | 2,075,708 | 933 | 2,225 |
| Mississippi | 1,797,114 | 343 | 5,239 |
| Missouri | 3,293,335 | 1,377 | 2,392 |
| Montana | 376,053 | 214 | 1,757 |
| Nebraska | 1,192,214 | 637 | 1,825 |
| Nevada | 81,875 | 45 | 1,819 |
| New Hampshire | 430,572 | 217 | 1,984 |
| New Jersey | 2,537,167 | 928 | 2,734 |
| New Mexico | 327,301 | 75 | 4,364 |
| New York | 9,113,614 | 4,270 | 2,135 |
| North Carolina | 2,206,287 | 430 | 5,131 |
| North Dakota | 577,056 | 179 | 3,224 |
| Ohio | 4,767,121 | 2,190 | 2,177 |
| Oklahoma | 1,657,155 | 326 | 3,243 |
| Oregon | 672,765 | 449 | 1,498 |
| Pennsylvania | 7,665,111 | 3,229 | 2,374 |
| Rhode Island | 542,610 | 278 | 1,952 |
| South Carolina | 1,515,400 | 360 | 4,209 |
| South Dakota | 583,888 | 200 | 2,919 |
| Tennessee | 2,184,789 | 560 | 3,920 |
| Texas | 3,896,542 | 940 | 4,145 |
| Utah | 373,351 | 208 | 1,795 |
| Vermont | 355,956 | 149 | 2,388 |
| Virginia | 2,061,612 | 449 | 4,592 |
| Washington | 1,141,990 | 611 | 1,869 |
| West Virginia | 1,221,119 | 375 | 3,256 |
| Wisconsin | 2,333,860 | 1,181 | 1,976 |
| Wyoming | 145,965 | 57 | 2,561 |
| Total | 91,972,266 | 38,876 | 2,366 |

An article captioned "Need for Dentists in Canada," published in the March number of *Oral Health,* shows in tabular form that there is a ratio of one dentist to 4,326 population in Canada, and goes on to say: "In the Province of Ontario there were one hundred more dentists in practice ten years ago than there are to-day—notwithstanding the large increase in population, as well as the greater demand for dental service. * * *"

If these figures show a real shortage of dentists in North America, what must be the dental needs of our far-off possessions, as shown by the following:

| | Population | Dentists | People to each Dentist |
|---|---|---|---|
| Hawaii | 191,909 | 23 | 8,344 |
| Philippine Islands | 8,000,000 | 56 | 142,857 |
| Porto Rico | 1,118,012 | 49 | 22,817 |
| Alaska | 64,356 | 34 | 1,893 |

To quote again from the *Oral Health,* respecting the shortage of dental practitioners: "This whole question is vital to the dental profession. It will be impossible to maintain the present dental standards * * * if the public demand for dental service be not met. The State has imposed certain rights and privileges upon the dental profession, and has entrusted to it the care of the dental needs of the public. The profession would be untrue to its trust if it did not make every possible effort to augment the present ranks of the profession to the end that the public may be well served. The solution lies largely in the hands of the dentist himself. Every dentist has a duty to perform in this matter, and should be ever alert to recommend the best type of young manhood to enter the profession and join in the dental campaign for good health which is being so successfully waged by the dental practitioner."—Editorial in May *Brief.*

